# Advanced Cross-Correlation Function Application to Identify Arterial Baroreflex Sensitivity Variations From Healthy to Diabetes Mellitus

**DOI:** 10.3389/fnins.2022.812302

**Published:** 2022-06-10

**Authors:** Shoou-Jeng Yeh, Chi-Wen Lung, Yih-Kuen Jan, Ben-Yi Liau

**Affiliations:** ^1^Section of Neurology and Neurophysiology, Cheng-Ching General Hospital, Taichung, Taiwan; ^2^Department of Creative Product Design, Asia University, Taichung, Taiwan; ^3^Rehabilitation Engineering Laboratory, Kinesiology and Community Health, Computational Science and Engineering, University of Illinois at Urbana-Champaign, Champaign, IL, United States; ^4^Department of Biomedical Engineering, Hungkuang University, Taichung, Taiwan

**Keywords:** baroreflex sensitivity, diabetes mellitus, autonomic neuropathy, blood pressure, heart rate, advanced cross-correlation function

## Abstract

Diabetes mellitus (DM) is a chronic disease characterized by elevated blood glucose levels, which leads over time to serious damage to the heart, blood vessels, eyes, kidneys, and nerves. DM is of two types–types 1 or 2. In type 1, there is a problem with insulin secretion, and in type 2–insulin resistance. About 463 million people worldwide have diabetes, and 80% of the majority live in low- and middle-income countries, and 1.5 million deaths are directly attributed to diabetes each year. Autonomic neuropathy (AN) is one of the common diabetic complications, leading to failure in blood pressure (BP) control and causing cardiovascular disease. Therefore, early detection of AN becomes crucial to optimize treatment. We propose an advanced cross-correlation function (ACCF) between BP and heart rate with suitable threshold parameters to analyze and detect early changes in baroreflex sensitivity (BRS) in DM with AN (DM+). We studied heart rate (HR) and systolic BP responses during tilt in 16 patients with diabetes mellitus only (DM−), 19 diabetes mellitus with autonomic dysfunction (DM+), and 10 healthy subjects. The ACCF analysis revealed that the healthy and DM groups had different filtered percentages of significant maximum cross-correlation function (CCF) value (*p* < 0.05), and the maximum CCF value after thresholds was significantly reduced during tilt in the DM+ group (*p* < 0.05). The maximum CCF index, a parameter for the phase between HR and BP, separated the healthy group from the DM groups (*p* < 0.05). Due to the maximum CCF index in DM groups being located in the positive range and significantly different from healthy ones, it could be speculated that BRS dysfunction in DM and AN could cause a phase change from lead to lag. ACCF could detect and separate DM+ from DM groups. This fact could represent an advantage of the ACCF algorithm. A common cross-correlation analysis was not easy to distinguish between DM− and DM+. This pilot study demonstrates that ACCF analysis with suitable threshold parameters could explore hidden changes in baroreflex control in DM+ and DM−. Furthermore, the superiority of this ACCF algorithm is useful in distinguishing whether AN is present or not in DM.

## Introduction

Diabetes mellitus (DM) is a chronic disease with elevated blood sugar in the human body. The WHO team indicates that DM is a widespread and challenging health problem due to its high growth prevalence and impact on national economies. It was also revealed that 463 million adults have the condition globally and that it has been steadily increasing over the years (Gregg et al., 2021). Uncontrolled DM with increased blood sugar (hyperglycemia) causes serious damage to the heart, blood vessels, eyes, kidneys, and nerves. Diabetic autonomic neuropathy (DAN) is a common complication of diabetes that involves the autonomic nervous system. Because all the human body organs, including the cardiovascular system, are regulated by the parasympathetic and sympathetic divisions of the ANS (Vinik et al., 2003), patients with DAN would have a high risk of inducing renal failure, heart disease, stroke, blindness, and death.

The ANS controls and modulates blood pressure (BP) and heart rate (HR) interaction rapidly through arterial baroreflex. A negative feedback system buffers fluctuations to maintain HR and BP during varying conditions in daily life (Heusser et al., 2005; Robertson et al., 2012; Kaufmann et al., 2020). Cardiovascular variability and baroreflex effectiveness may be influenced by age, gender, reflex, humoral, behavioral, and environmental factors (Laitinen et al., 1998; Lanfranchi and Somers, 2002). Baroreflex measurement and assessment could be used as an index for circulatory regulation at the sinoatrial node (Lanfranchi and Somers, 2002; Jíra et al., 2006; La Rovere et al., 2008; Dutra-Marques et al., 2021; Heusser et al., 2021). A previous study reviewed approaches to measure baroreflex sensitivity (BRS), using the sequence method, cross-correlation method, cross-spectral method, synchronization index, cross multiscale entropy, joint symbolic dynamics, similarity index, etc., in DM. The study revealed that DM has baroreflex impairment with reduced baroreflex response and prolonged baroreflex (Javorka et al., 2011; Xiao et al., 2019; Cseh et al., 2020). Abnormal baroreflex function could indicate autonomic cardiovascular imbalance early (Ziegler et al., 2018; Cseh et al., 2020). Present BRS analysis methods based on HR and BP analysis might not be able to observe the sympathetic baroreflex component by its nature (Persson et al., 2001; Swenne, 2013; Kawada et al., 2021). Therefore, impaired baroreflex function could not always be detected due to this limitation or other reasons (Laude et al., 2004).

The cross-correlation function (CCF) estimates the relationship level between the two signals. It can be used to find hidden temporal similarities (Derrick and Thomas, 2004). This method has been applied to characterize autonomic and baroreflex control (Westerhof et al., 2004; Silvani et al., 2011). For instance, the method (cross-correlation BRS, xBRS) was applied to test EUROBAVAR data, including patients with a heart transplant and DAN. As a result, a different baroreflex delay was measured in patients compared with healthy subjects (Westerhof et al., 2004). Furthermore, CCF has been used successfully to evaluate cerebral autoregulation to find differences between healthy individuals and patients (Czosnyka et al., 1996; Steinmeier et al., 1996; Panerai et al., 2000, 1996; Chiu and Yeh, 2001). Recently, cross wavelet analysis was applied to follow BRS change in the time–frequency domain to understand sympathetic participation (de Boer and Karemaker, 2019). However, some non-stationary factors (e.g., weak baroreflex, frequent arrhythmias, respiratory, etc.) and unknown peripheral noise could affect the accuracy of the BRS estimation results. Therefore, a new baroreflex assessing procedure was needed to increase reliability (Persson et al., 2001; Swenne, 2013; Kawada et al., 2021; Pinheiro et al., 2021; Yamasaki et al., 2021). The purpose of this study is to propose an advanced cross-correlation function (ACCF) with suitable threshold parameters to reduce noise and inaccuracy in estimating baroreflex properties. We hypothesize that this approach (ACCF) can detect early changes in BRS in the DM group.

## Materials and Methods

### Subjects and Measurement

In total, 35 age-matched patients with DM were enrolled in this study. There were 16 patients without autonomic neuropathy (AN) (DM−, 55.63 ± 15.49 years) and 19 patients with DM with AN (DM+, 65.87 ± 11.11 years). In addition, 10 healthy subjects (57.40 ± 8.41 years) were recruited. Medication was withdrawn during the study period. The AN was determined by the clinical autonomic reflex tests (R–R variation, Valsalva maneuver, and postural BP testing) (American Diabetes Association, 1992). The patients had no other cardiovascular disease and no significant BP difference (*p* > 0.05) among groups in the supine position. This study was approved by the Research Ethics Committee of Cheng-Ching General Hospital, Taiwan. Subjects were examined on a tilting table with a motor-driven change from horizontal supine to upright positions. Data acquisition was started after a 10 min relaxation period in the resting position. Then, the subject was head-up tilted to 75° within 4 s to induce BP fluctuation. A continuous arterial blood pressure (ABP) signal was acquired at 60 Hz sample frequency for 5 min by Finapres (Model 2300, Ohmeda, Englewood, CO, United States) during supine and head-up tilt positions. Signals were analyzed with their own developed system using the software—LabVIEW language (Chiu and Yeh, 2001; Chiu et al., 2005).

**FIGURE 1:**
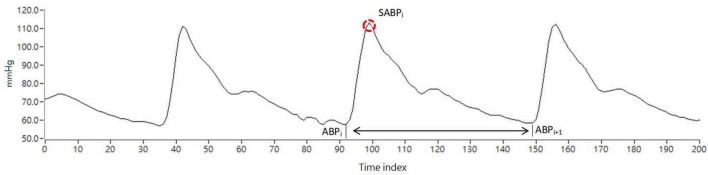
Representation of the ABP signal, the red circle is the blood pressure wave peak.

Figure 1 shows the representation of the ABP signal. While the ABP pulse signal was acquired continuously by Finapres, SABPi (*i*th systolic arterial blood pressure) is the waveform peak through time index in the *i*th ABP pulse beat. Therefore, SABPi is the maximum BP value calculated by the *i*th pulse beat. The systolic arterial blood pressure (SABP) value was calculated using each pulse as follows:


$${\text{SABP}}_{i}=\max\left({\text{ABP}}_{i},{\mathrm{ABP}}_{i+1}\right)$$

Simultaneously, systolic BP was aligned with the R–R interval, and instantaneous HR was derived from the point at which the systolic BP event occurred. In the previous studies, beat-to-beat BP and HR could be recorded by photoplethysmography. The accuracy of beat-to-beat non-invasive measurement *via* finger arterial pressure using Finpres was confirmed (Novak et al., 1994; Buclin et al., 1999).

### Advanced Cross-Correlation Function Estimation

#### Cross-Correlation Function Estimation for Baroreflex Sensitivity

Cross-correlation is a method to assess the similarity degree of two series data sets. The cross-correlation function (CCF) could provide a measure of association between time-series signals (Derrick and Thomas, 2004). In this study, the SABP signal and the beat-to-beat HR signal as the input data for estimation. The relation and phase between the SABP and the HR signals can be used to evaluate the baroreflex function. The cross-correlation and phase between BP and HR depend on the autonomic baroreflex control. Therefore, it could explore changes in baroreflex mechanisms for health and disease.

Let the cross-correlation function be expressed as *CCF(k)*, *W* is the length of the window, *k* is the number of peak-to-peak displacement points, and *N* is the total signal length. Assume that the SABP and HR signals are represented as *f(n)* and *g(n)*, respectively. To assess the autonomic nervous system in specific frequency bands, *f(n)* and *g(n)* signals were bandpass filtered in low frequency (LF) ranges before applying the CCF. Where the LF range is 0.07–0.15 Hz, assume that bandpass filtered *f(n)* and *g(n)* signals are $\hat{f}\,\left(n\right)$and $\hat{g}\,\left(n\right)$, respectively. The *CCF* between $\hat{f}\,\left(n\right)$and $\hat{g}\,\left(n\right)$ can be calculated as follows:


(1) $$C\,C\,F_{i}\,\left(k\right)=\frac{R_{\hat{f}\,\hat{g}}^{i}\,\left(k\right)}{{\left[R_{\hat{f}\,\hat{f}}^{i}\,\left(0\right)\,R_{\hat{g}\,\hat{g}}^{i}\,\left(0\right)\right]}^{\frac{1}{2}}}\,\ldots \,k=0,\pm 1,\pm 2,\cdots ,i=1\,\mathrm{to}\,\text{N–W}+1\,$$

where $R_{\hat{f}\,\hat{g}}^{i}\,\left(k\right)$ is an estimation of the cross-covariance in the *i*th time window and defined as


(2) $$R_{\hat{f}\,\hat{g}}^{i}\,\left(k\right)=\left\{ \begin{array}{c} \frac{1}{W}\,\sum_{j=i}^{i+W}\hat{f}\,\left(j\right)\,\hat{g}\,\left(j+k\right),\,k=0,\,1,\,2,\cdots \\ \frac{1}{W}\,\sum_{j=i}^{i+W}\hat{f}\,\left(j-k\right)\,\hat{g}\,\left(j\right),\,k=0,-1,-2,\cdots \end{array}\right.$$

$$\text{Also}\,R_{\hat{f}\,\hat{f}}^{i}\,\left(0\right)=\frac{1}{W}\,\sum_{j=i}^{i+W}{\left[\hat{f}\,\left(j\right)\right]}^{2},\text{and}\,R_{\hat{g}\,\hat{g}}^{i}\,\left(0\right)=\frac{1}{W}\,\sum_{j=i}^{i+W}{\left[\hat{g}\,\left(j\right)\right]}^{2}.$$

where *N* is the total number of cardiac cycles, *W* is the window width, and *k* is the time lag. In this study, +*k* means that HR follows BP. *CCF*_*i*_(⋅) is the outcome of *CCF* between $\hat{f}\,\left(n\right)$ and $\hat{g}\,\left(n\right)$ in the *i*th time window.

#### Advanced Cross-Correlation Function Estimation Using the Thresholding

As described in section “*Cross-Correlation Function* Estimation for Baroreflex Sensitivity,” the original *CCF* estimation has *N* = 256, *W* = 64, and no other threshold following the *CCF* estimation. Figure 1 shows typical 2D figures using the *CCF* estimation. Due to *i* = 1 to *N* − *W*+1, there are 193 *CCF* curves in this study. A typical 2D representative figure is shown in Figure 1.

In *ACCF* estimation, we set thresholding parameters for *k* to improve some limitations and filter some noise to avoid a result bias. The thresholding was as follows:

Followed the *CCF* estimation, we set *N* = 256, *W* = 64, and −5 ≦ *k* ≦ 5.

The *ACCF* with thresholding between $\hat{f}\,\left(n\right)$and $\hat{g}\,\left(n\right)$ can be calculated as follows:


(3) $${\mathrm{ACCF}}_{i}\left(k\right)=\frac{R_{\hat{f}\,\hat{g}}^{i}\,\left(k\right)}{{\left[R_{\hat{f}\,\hat{f}}^{i}\,\left(0\right)\,R_{\hat{g}\,\hat{g}}^{i}\,\left(0\right)\right]}^{\frac{1}{2}}}>threshold,k=0,+1,+2,+3,+4,+5$$

where the threshold = 0, 0.3, 0.5, 0.7.

**FIGURE 2:**
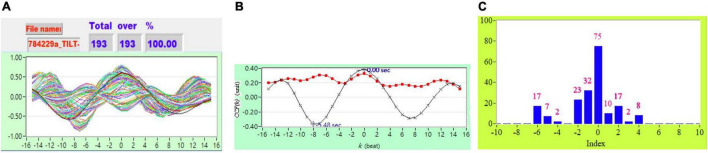
**(A)** Representative plot of all *CCF* curves without thresholding, **(B)** mean *CCF* value (*x*) and SD (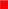), and **(C)** distribution bar chart of maximum *CCF* index during tilt in a healthy subject.

**FIGURE 3:**
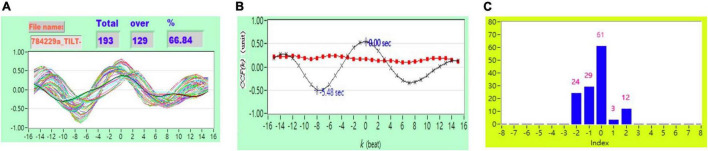
**(A)** Representative plot of *CCF* curves with thresholding [–5 ≦ *k* ≦ 5 and *CCF(k)* > 0], **(B)** mean *CCF* value (*x*) and SD (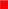), and **(C)** distribution bar chart of maximum *CCF* index during tilt in a healthy subject.

**FIGURE 4:**
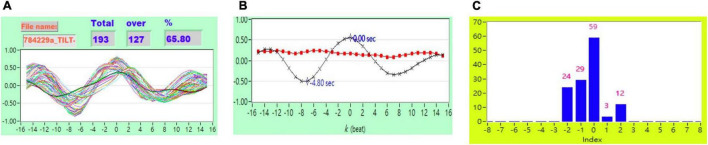
**(A)** Representative plot of *CCF* curves with thresholding [–5 ≦ *k* ≦ 5 and *CCF(k)* > 0.3], **(B)** mean *CCF* value (*x*) and SD (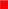), and **(C)** distribution bar chart of maximum *CCF* index during tilt in a healthy subject.

**FIGURE 5:**
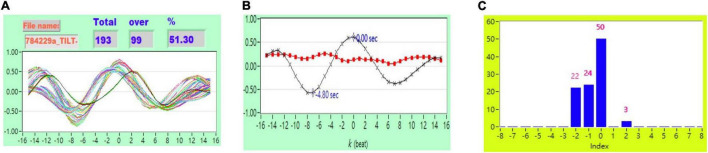
**(A)** Representative plot of *CCF* curves with thresholding [–5 ≦ *k* ≦ 5 and *CCF(k)* > 0.5], **(B)** mean *CCF* value (*x*) and SD (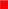), and **(C)** distribution bar chart of maximum *CCF* index during tilt in a healthy subject.

**FIGURE 6:**
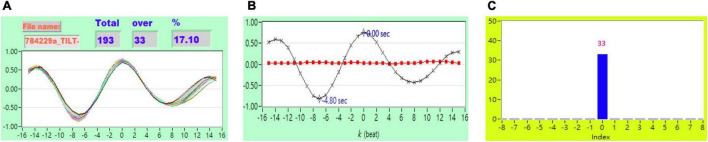
**(A)** Representative plot of *CCF* curves with thresholding [–5 ≦ *k* ≦ 5 and *CCF(k)* > 0.7] **(B)** mean *CCF* value (*x*) and SD (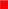), and **(C)** distribution bar chart of maximum *CCF* index during tilt in a healthy subject.

Figure 2 shows the representative plot of all *CCF* curves without threshold. Figures 3–6 are the representative plot of all *CCF* curves with different thresholds, respectively.

## Results

### The Filtered Proportion of Maximum *Cross-Correlation Function* Value

**FIGURE 7:**
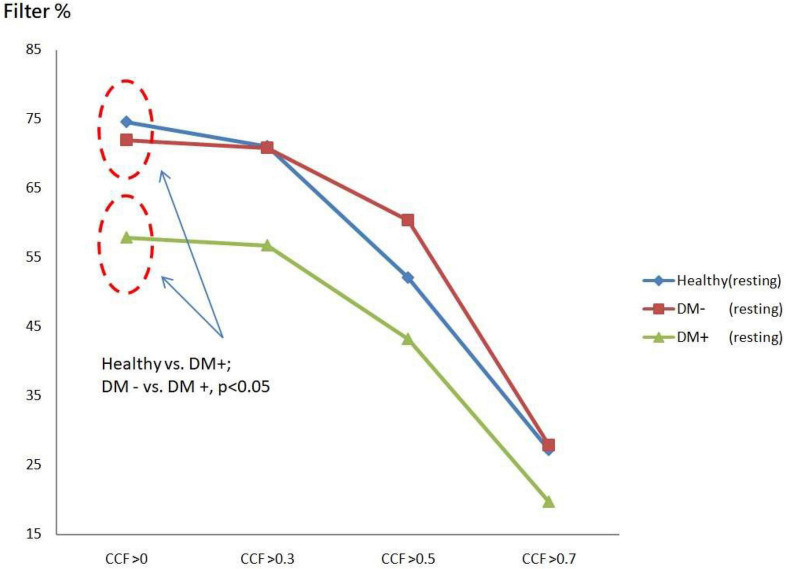
The plot of filter % with different thresholds by *ACCF* results in the supine position. Filtered maximum *CCF* value (% to all *CCF* values) using the threshold *CCF* > 0, 0.3, 0.5, and 0.7 in resting position in healthy (blue), diabetes mellitus (DM–, red), and DM with autonomic neuropathy (DM+, green). Threshold *CCF* > 0 allowed to differentiate healthy, DM–, and DM+.

The filtered value was the proportion of *CCF* values that passed the threshold set for all *CCF* values without thresholds. Figure 7 shows *ACCF* results for the supine position. The filtered value was higher in the healthy group than in the DM groups when the *CCF* threshold was set to be greater than 0 (*CCF* > 0). This threshold of *CCF* > 0 permits separating the healthy group from DM groups (*p* < 0.05) in the resting position. The two DM groups, DM− and DM+, also showed significantly different filtered values (*p* < 0.05). It revealed the effect of AN on DM that the relation level between BP and HR by the maximum *CCF* value. The filtered amount of *CCF* values in the DM+ group always keeps the lower percentage values at every threshold.

**FIGURE 8:**
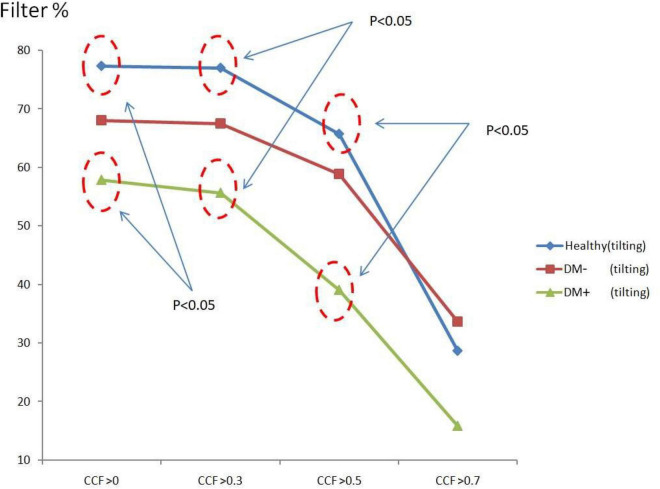
The plot of filter % with different thresholds by *ACCF* results in a tilting position. *ACCF* results of tilt. Filtered maximum CCF value (% to all CCF values) using the threshold *CCF* > 0, 0.3, 0.5, and 0.7 in resting position in healthy (blue), diabetes mellitus (DM–, red), and DM with autonomic neuropathy (DM+, green). CCF thresholds of >0, >0.3, and >0.5 allowed for the differentiation of healthy and DM+ individuals.

Figure 8 shows the *ACCF* results of tilt. The filtered value (% of all CCF values) was higher in the healthy than those in the DM+ group when the *CCF* threshold was set at *CCF* > 0, *CCF* > 0.3, and *CCF* > 0.5. The healthy group could be significantly separated from the DM+ group (*p* < 0.05).

The filtered *CCF* values tended to be lower with the higher threshold in healthy and DM groups using the threshold setting. In the DM+ group, the least filtered percentage of *CCF* values was always kept at every different threshold. It could show a more significant difference between the healthy group and DM+ group in an upright position.

### Maximum Cross-Correlation Function Index With Threshold

**FIGURE 9:**
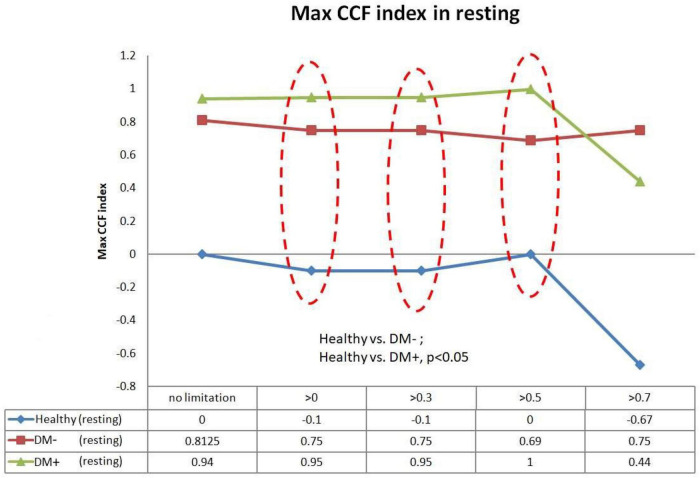
The plot of the maximum *CCF* index with different thresholds by *ACCF* results in the supine position. *ACCF* phase analysis during resting supine. Filtered maximum CCF index in healthy (blue), diabetes mellitus (DM–, red), and DM with autonomic neuropathy (DM+, green). When the threshold setting was greater than 0, 0.3, and 0.5, the filtered maximum CCF index could significantly separate the healthy group from the DM– and DM+ group (*p* < 0.05). It also indicates that the phase between HR and BP was prolonged in DM.

Figure 9 shows the filtered maximum *CCF* index in the resting supine. When the threshold setting for maximum *CCF* value is greater than 0, 0.3, and 0.5, the filtered maximum *CCF* index could significantly separate the healthy group from the DM group (*p* < 0.05). It also indicated that the phase between HR and BP was prolonged in DM. In addition, the two DM groups could be separated significantly (*p* < 0.05) when the threshold was set to 0, 0.3, and 0.5.

**FIGURE 10:**
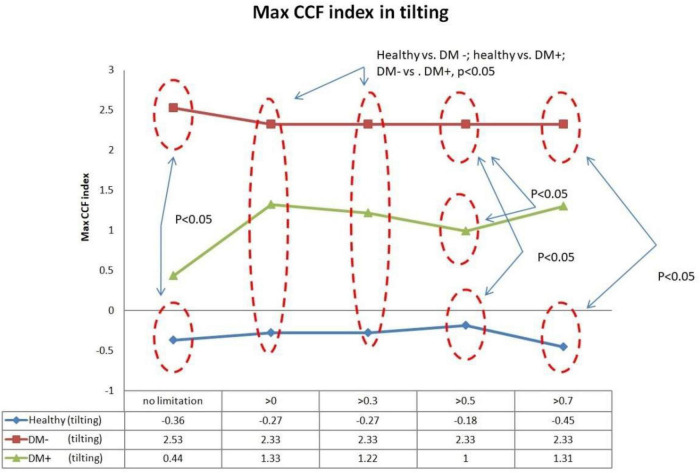
The plot of the maximum *CCF* index with different thresholds by *ACCF* results in a tilting position. *ACCF* phase analysis during tilt. Filtered maximum *CCF* index in healthy (blue), diabetes mellitus (DM–, red), and DM with autonomic neuropathy (DM+, green). When the threshold setting was set greater than 0, 0.3, 0.5, and 0.7, the filtered maximum *CCF* index could significantly separate the healthy group from the DM– and DM+ groups (*p* < 0.05). In addition, the two DM groups could be separated (*p* < 0.05) when the threshold was set to 0, 0.3, and 0.5.

Figure 10 shows the filtered maximum *CCF* index during tilt. The filtered maximum *CCF* index value was greater during the upright position when the threshold was set at greater than 0, 0.3, 0.5, and 0.7. The max *CCF* index could completely separate the healthy group from the DM (*p* < 0.05). In addition, the two DM groups could be separated (*p* < 0.05) when the threshold was set to 0, 0.3, and 0.5.

### Comparison of Maximum Cross-Correlation Function Value in Response to Head-up Tilting

**FIGURE 11:**
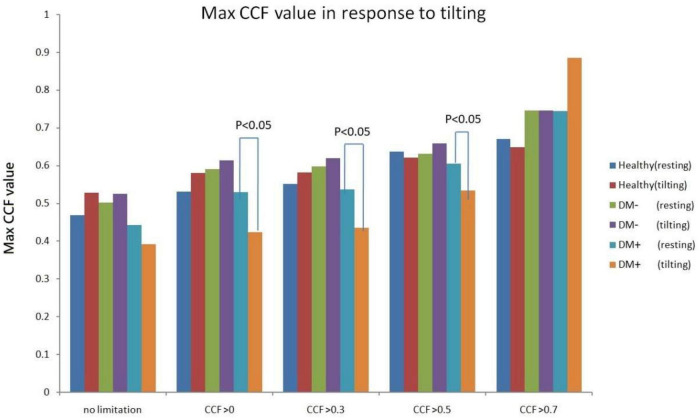
Maximum *CCF* value response to tilt in healthy (red), diabetes mellitus (DM–, purple), and DM with autonomic neuropathy (DM+, orange) after thresholding of *CCF* > 0, 0.3, 0.5, and 0.7.

Figure 11 shows the estimated CCF value after filter during tilt. The results indicate that when the threshold for *CCF* was set greater than 0, 0.3, and 0.5, the estimate of maximum *CCF* values of the DM+ group was reduced significantly (*p* < 0.05) in response to tilting, while *CCF* did not change in healthy.

**FIGURE 12:**
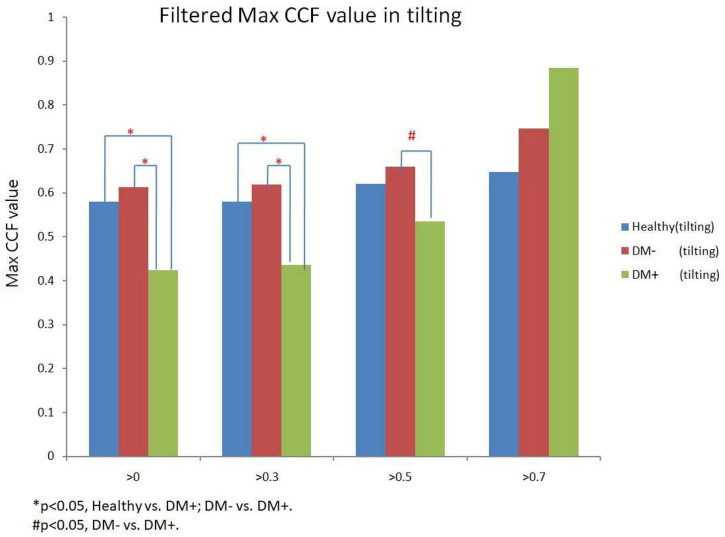
The filtered maximum *CCF* value in healthy (blue), diabetes mellitus (DM–, red), and DM with autonomic neuropathy (DM+, green) using the threshold *CCF* > 0, 0.3, 0.5, and 0.7 during tilting position. The symbol “*” and “#” mean the *p*-value smaller than 0.05 in *t*-test.

Figure 12 shows the filtered maximum *CCF* value after thresholding during tilt position. When the threshold was greater than 0, 0.3, and 0.5, the maximum *CCF* values were significantly lower in DM+ (*p* < 0.05) than in the DM− group. Moreover, when the threshold was greater than 0 and 0.3, the maximum *CCF* values of DM+ were significantly lower (*p* < 0.05) than those in the healthy group. Thresholding *CCF* > 0, 0.3, and 0.5 during tilt distinguished between DM+ and DM−.

## Discussion

To the best of our knowledge, this is the first study to investigate baroreflex performance and differences using the *ACCF* to observe progress in baroreflex dysfunction from healthy to DAN. The thresholding for maximum CCF value showed that the DM+ group showed the least filtered percentage relative to all CCF values. In addition, the percentage of filtered value in the DM+ group was significantly different from the healthy group. That could indicate that the baroreflex function mediated the relationship between BP and HR is increased more than those in healthy and DM− groups. This difference could not be found using a common CCF analysis method without a threshold (Figure 10).

In addition, the maximum CCF value after thresholding is significantly reduced during tilt in the DM+ group. In clinical practice, AN diagnosis requires a series of complex tests. It is worth noting that during upright, maximum CCF values after thresholding were significantly different in the DM+ group from those in the healthy and DM− groups. This fact could represent an advantage of the ACCF algorithm to distinguish DM+ or DM−. We demonstrated that setting the threshold for maximum CCF value in the estimation process could differentiate between DM+ and healthy groups and even DM+ and DM− groups. It is known that the BRS is reduced in patients with DM and cannot counteract BP fluctuations effectively. Furthermore, patients with DM+ are unable to regulate their HR, blood vessel tone, and other parameters due to baroreflex dysfunction, which raises the risk of cardiovascular disease (Kuusela et al., 2002; Yu et al., 2011; Fuchs and Ehelton, 2020).

Furthermore, we could show that the phase between BP and HR, determined by the maximum *CCF* index in the *ACCF* estimation, is different in healthy and DM groups. A negative lag represents a phase-lead characteristic (BP leads HR) (Chiu and Yeh, 2001; Chiu et al., 2005). Recently, de Boer and Karemaker (2019) assessed BRS by cross wavelet analysis and head-up tilt. The results enabled the estimation of cross-spectra and derived quantities of BRS during time and frequency conditions. Some researchers assumed that baroreflex dysfunction causes a reduction of phase-lead to nearly zero time delay (Steinmeier et al., 1996). Our results confirm the previous support. In this study, Figures 9, 10 show the maximum *CCF* index of *ACCF* estimation results, which indicates the maximum *CCF* index in the healthy group, was in the negative range for both resting and tilting positions. Thus, the analysis results of the healthy and DM+ groups were confirmed by a previous report (Westerhof et al., 2004), and it may be standard for normal BRS function.

Interestingly, the maximum *CCF* index in DM groups was located in the positive range and was significantly different from those in the healthy group. We speculated that BRS dysfunction in DM and AN could cause a change from phase-lead to phase-lag. Furthermore, in line with previous studies, baroreflex response was reduced, and the delay was prolonged (Javorka et al., 2011; Xiao et al., 2019; Cseh et al., 2020), which supported the assumption. Therefore, we proposed an advanced cross-correlation function to estimate BRS in healthy and DM groups. Our results revealed that significant differences between the groups would be shown when the threshold setting for maximum CCF value was 0–0.5. It might be suggested that the proposed thresholding of maximum CCF from 0 to 0.5 improves the separation of DM+ and DM−.

## Conclusion

Advanced CCF analysis with suitable thresholding improves the detection of changes in DM’s BRS. The new method could explore hidden changes in circulatory system component characteristics and BRS performance and provide new insight and risk prediction in patients with DM.

## Data Availability Statement

The raw data supporting the conclusions of this article will be made available by the authors, without undue reservation.

## Ethics Statement

The studies involving human participants were reviewed and approved by the Research Ethics Committee of Cheng-Ching General Hospital, Taiwan. The patients/participants provided their written informed consent to participate in this study.

## Author Contributions

B-YL and S-JY: conceptualization and methodology. B-YL and Y-KJ: formal analysis. B-YL, C-WL, and Y-KJ: writing. B-YL: project administration. All authors have read and agreed to the published version of the manuscript.

## Conflict of Interest

The authors declare that the research was conducted in the absence of any commercial or financial relationships that could be construed as a potential conflict of interest.

## Publisher’s Note

All claims expressed in this article are solely those of the authors and do not necessarily represent those of their affiliated organizations, or those of the publisher, the editors and the reviewers. Any product that may be evaluated in this article, or claim that may be made by its manufacturer, is not guaranteed or endorsed by the publisher.
